# Delayed-Onset Loiasis: A Case of Loa loa Infection Diagnosed Six Years Post-exposure

**DOI:** 10.7759/cureus.86135

**Published:** 2025-06-16

**Authors:** Halle N Root, Diana Bueno, Nicholas Bathurst, Michael Wieting

**Affiliations:** 1 Medical School, Lincoln Memorial University, DeBusk College of Osteopathic Medicine, Knoxville, USA; 2 Medical School, Lincoln Memorial University, DeBusk College of Osteopathic Medicine, Harrogate, USA; 3 Physical Medicine and Rehabilitation, Osteopathic Manipulative Medicine, Lincoln Memorial University, DeBusk College of Osteopathic Medicine, Knoxville, USA

**Keywords:** albendazole treatment, delayed diagnosis, filarial infection, loa loa, loiasis, ocular parasite, parasitic disease

## Abstract

Loiasis, caused by *Loa loa*, is a filarial infection endemic to Central and West Africa, increasingly recognized in non-endemic regions due to global migration and travel. Diagnosis in non-endemic regions can be challenging due to delayed symptom onset. We report a case of a 40-year-old woman presenting with ocular discomfort and a migrating subcutaneous parasite six years after residing in Gabon. Laboratory evaluation revealed eosinophilia and microfilariae on peripheral blood smear, confirming loiasis. Initial treatment with albendazole led to inflammatory symptoms, highlighting the risk of post-treatment reactions. This case underscores the importance of recognizing loiasis in patients with remote travel history, the need for careful microfilarial burden assessment, and multidisciplinary management to optimize treatment outcomes.

## Introduction

Loiasis is a parasitic infection caused by the filarial nematode* Loa loa*, transmitted by the chrysops fly. Although not currently listed as a neglected tropical disease (NTD) by the World Health Organization (WHO), its growing public health impact, particularly in Central and West Africa, has prompted calls for its inclusion due to diagnostic difficulties, treatment-associated risks, and its high prevalence in endemic regions [1,2]. An estimated 14.4 million people live in high-risk areas where the prevalence of eye worm history is greater than 40%, with most cases concentrated in countries such as Gabon, Cameroon, and the Democratic Republic of the Congo [2]. With increased global travel and migration, cases are now increasingly reported in non-endemic areas, including Europe and North America [3]. Clinical manifestations vary and may appear years after exposure, which may delay recognition and complicate diagnosis in non-endemic settings. This case highlights the diagnostic and treatment challenges of delayed-onset loiasis in a patient presenting in the United States.

## Case presentation

A 40-year-old African American female patient who resides in Ocala, Florida, presented to the emergency department (ED) at AdventHealth Ocala with complaints of left eye discomfort and suspicion of a parasitic infection in the eye in October 2024. The patient’s past medical history was notable for having resided in Gabon, Central Africa. She lived in Gabon six years ago for approximately 1.5 years and had not traveled outside or within the United States since then. 

**Figure 1:**
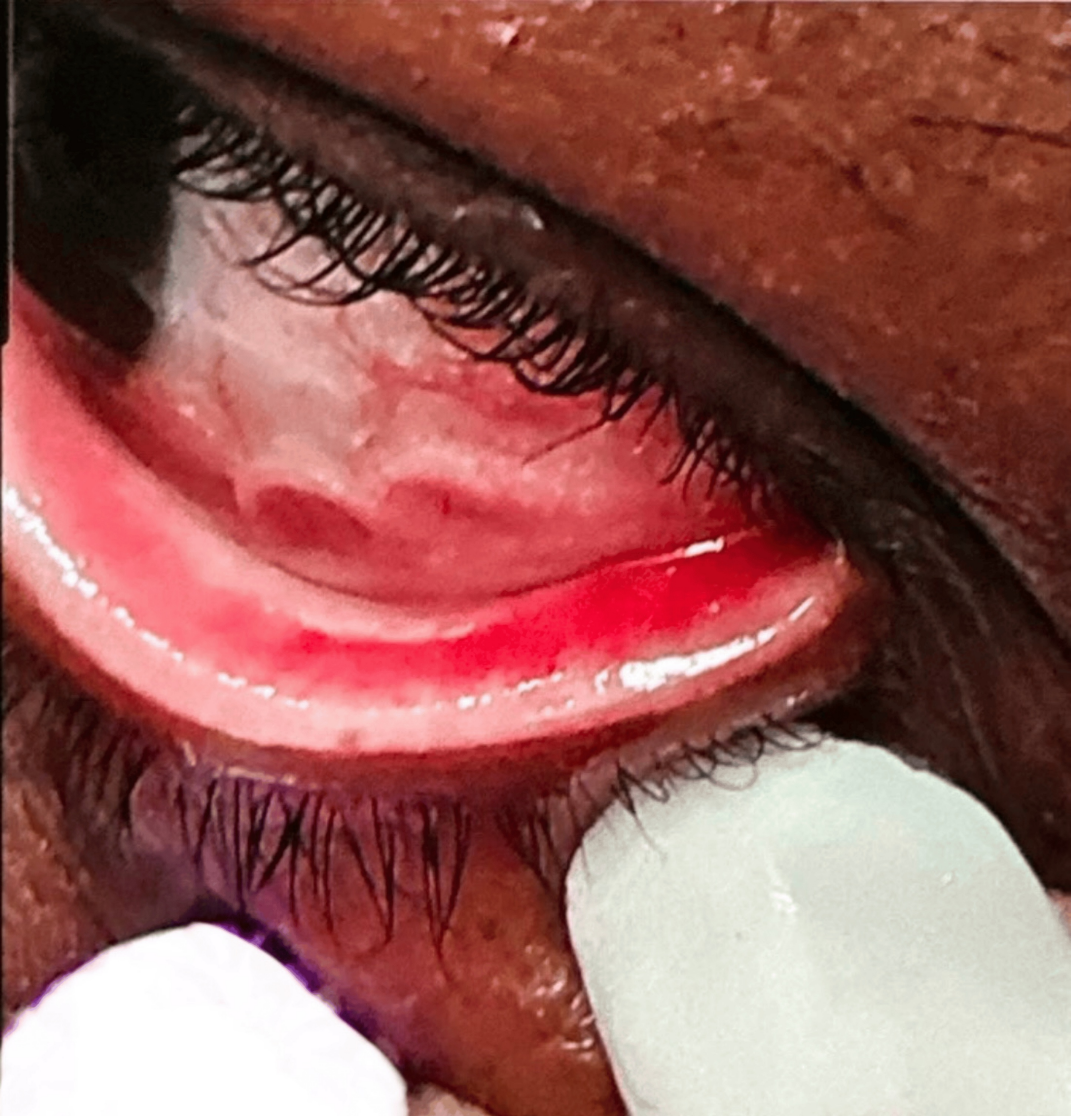
Subconjunctival Loa loa Worm Observed in Patient's Left Eye

Upon physical examination of the left eye, the conjunctiva was erythemic but without periorbital swelling or lid edema. All extraocular muscles were intact, and pupils were equal and reactive to light bilaterally. Fundoscopic exam was unremarkable, with no evidence of retinal or optic disc involvement. There was no evidence of a worm in her sclera; however, a small worm-like organism was identified actively moving over the patient’s upper eyelid. The right eye was unremarkable. The patient shared images from her personal phone revealing a subconjunctival parasite in her left eye (Figure 1).

A CT scan of the facial bones and head revealed no acute findings. The soft tissues demonstrated symmetric and intact bilateral globes and intraorbital contents, with no evidence of extraocular muscle abnormality. A Wood's lamp examination did not reveal any corneal abrasion or globe interruption. The patient’s vital signs were stable, and the rest of the physical exam was unremarkable. She was discharged from the ED with a diagnosis of loiasis and a prescription for albendazole 100 mg to be taken for 21 days. This dosage was selected as a well-tolerated, stepwise strategy to reduce microfilarial burden prior to potential definitive therapy. Additionally, topical erythromycin was prescribed to serve as a prophylactic antibiotic and lubricant for her eye. She was advised to follow up with outpatient infectious disease (ID) and ophthalmology specialists.

Despite initiating treatment, she returned to the ED eight days later with resolution of her initial eye symptoms but new-onset swelling at various locations on her face and the back of her neck, as well as new-onset numbness, pain, and scalp pruritus. She had not yet followed up with specialists. A CT scan of the head and neck revealed no acute pathology, and blood cultures showed no growth. Laboratory evaluation demonstrated a significantly elevated eosinophil count (23.1%). Examination findings for preauricular and submandibular lymph nodes were not documented in the medical record. 

**Figure 2:**
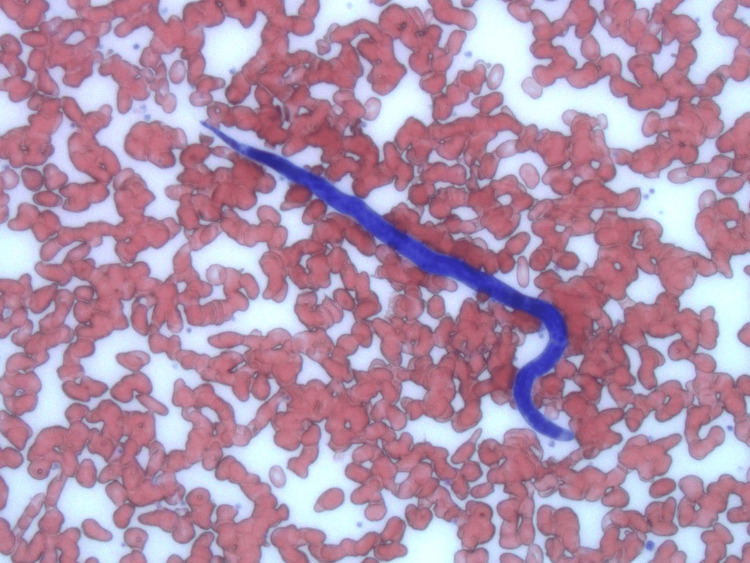
Peripheral Blood Smear Showing a Motile Loa loa Microfilaria with Characteristic Morphology

To reconfirm the diagnosis of loiasis, a peripheral blood smear was performed during midday hours (10:00 AM to 2:00 PM) when microfilariae are typically present in higher concentrations. The smear revealed motile microfilariae with morphological features characteristic of *Loa loa*, confirming the diagnosis [2] (Figure 2).

She was informed that her symptoms were likely the result of antiparasitic therapy. The patient was discharged with a new prescription for albendazole 400 mg for 21 days and advised to follow up with ID and ophthalmology. She later reported resolution of symptoms.

## Discussion

This case exemplifies the diagnostic challenges of delayed-onset loiasis in a non-endemic region. *Loa loa* is unique among human filarial parasites for its migratory behavior through subcutaneous tissues and conjunctiva. It is transmitted by* Chrysops silacea* and *Chrysops dimidiata* flies endemic to Central and West African rainforests [3].

Two forms of the parasite contribute to clinical manifestations: adult worms that migrate across the eyes and subcutaneous tissues, and microfilariae that circulate in the blood and trigger eosinophilia. Symptom onset averages 3.8 years post-infection [4], though our patient presented after six years. Temporary residents of endemic areas are more prone to systemic inflammatory symptoms, whereas endemic natives more often experience visible ocular migration [5].

Diagnosis requires high clinical suspicion, especially in patients with travel history. Peripheral blood smears collected during peak microfilaremia (midday) remain the gold standard. Typical *Loa loa* microfilariae measure 231-250 µm with irregularly arranged nuclei and a small headspace [6,7].

Loiasis can present asymptomatically or with complications including Calabar swellings, pruritus, encephalopathy, and ocular discomfort. High microfilarial burdens pose treatment risks, particularly with ivermectin or diethylcarbamazine (DEC), which have been associated with encephalopathy [8,9]. Even asymptomatic microfilaremia has been associated with increased mortality risk [10]. Albendazole is used to reduce microfilarial burden safely. Inflammatory symptoms post-treatment, as observed in this patient, are linked to elevated IL-5 levels, especially in temporary residents [11].

## Conclusions

This case illustrates the importance of considering loiasis in patients presenting years after travel to endemic regions. Timely diagnosis, appropriate diagnostic techniques such as peripheral smear timing, and a stepwise therapeutic approach, including the use of albendazole before DEC or ivermectin, are crucial for minimizing complications. This case also highlights the importance of recognizing inflammatory reactions post-treatment and underscores the need for multidisciplinary follow-up to ensure optimal outcomes.

## References

[1] Zouré HG, Wanji S, Noma M, Amazigo UV, Diggle PJ, Tekle AH, Remme JH (2011). The geographic distribution of Loa loa in Africa: results of large-scale implementation of the Rapid Assessment Procedure for Loiasis (RAPLOA). PLoS Negl Trop Dis.

[2] (2025). Centers for Disease Control and Prevention. Clinical testing and diagnosis for loiasis. https://www.cdc.gov/filarial-worms/hcp/diagnosis-testing/index.html.

[3] Antinori S, Schifanella L, Million M (2012). Imported Loa loa filariasis: three cases and a review of cases reported in non-endemic countries in the past 25 years. Int J Infect Dis.

[4] Kelly-Hope L, Paulo R, Thomas B, Brito M, Unnasch TR, Molyneux D (2017). Loa loa vectors Chrysops spp.: perspectives on research, distribution, bionomics, and implications for elimination of lymphatic filariasis and onchocerciasis. Parasit Vectors.

[5] Herrick JA, Metenou S, Makiya MA, Taylar-Williams CA, Law MA, Klion AD, Nutman TB (2015). Eosinophil-associated processes underlie differences in clinical presentation of loiasis between temporary residents and those indigenous to Loa-endemic areas. Clin Infect Dis.

[6] Mathison BA, Couturier MR, Pritt BS (2019). Diagnostic identification and differentiation of microfilariae. J Clin Microbiol.

[7] Marie C, Petri WA (2025). MSD Manual Professional Edition. Loiasis - infectious diseases. Loiasis - Infectious Diseases. MSD Manual Professional Edition.

[8] Boussinesq M, Gardon J, Gardon-Wendel N, Kamgno J, Ngoumou P, Chippaux JP (1998). Three probable cases of Loa loa encephalopathy following ivermectin treatment for onchocerciasis. Am J Trop Med Hyg.

[9] Boussinesq M, Gardon J, Gardon-Wendel N, Chippaux JP (2003). Clinical picture, epidemiology and outcome of Loa-associated serious adverse events related to mass ivermectin treatment of onchocerciasis in Cameroon. Filaria J.

[10] Burger G, Adamou R, Kreuzmair R (2024). Eosinophils, basophils and myeloid-derived suppressor cells in chronic Loa loa infection and its treatment in an endemic setting. PLoS Negl Trop Dis.

[11] Ramharter M, Butler J, Mombo-Ngoma G, Nordmann T, Davi SD, Zoleko Manego R (2024). The African eye worm: current understanding of the epidemiology, clinical disease, and treatment of loiasis. Lancet Infect Dis.

